# Absence of Sex-Contingent Gaze Direction Aftereffects Suggests a Limit to Contingencies in Face Aftereffects

**DOI:** 10.3389/fpsyg.2015.01829

**Published:** 2015-12-01

**Authors:** Nadine Kloth, Gillian Rhodes, Stefan R. Schweinberger

**Affiliations:** ^1^Australian Research Council Centre of Excellence in Cognition and its Disorders, School of Psychology, The University of Western Australia, Perth, WA, Australia; ^2^DFG Research Unit Person Perception, Friedrich Schiller University of Jena, Jena, Germany; ^3^Department of Psychology, Friedrich Schiller University of Jena, Jena, Germany

**Keywords:** perceptual adaptation, gaze direction aftereffect, face adaptation, simultaneous opposite aftereffects, contingent aftereffects, face sex

## Abstract

Face aftereffects (e.g., expression aftereffects) can be simultaneously induced in opposite directions for different face categories (e.g., male and female faces). Such aftereffects are typically interpreted as indicating that distinct neural populations code the categories on which adaptation is contingent, e.g., male and female faces. Moreover, they suggest that these distinct populations selectively respond to variations in the secondary stimulus dimension, e.g., emotional expression. However, contingent aftereffects have now been reported for so many different combinations of face characteristics, that one might question this interpretation. Instead, the selectivity might be generated during the adaptation procedure, for instance as a result of associative learning, and not indicate pre-existing response selectivity in the face perception system. To alleviate this concern, one would need to demonstrate some limit to contingent aftereffects. Here, we report a clear limit, showing that gaze direction aftereffects are not contingent on face sex. We tested 36 young Caucasian adults in a gaze adaptation paradigm. We initially established their ability to discriminate the gaze direction of male and female test faces in a pre-adaptation phase. Afterwards, half of the participants adapted to female faces looking left and male faces looking right, and half adapted to the reverse pairing. We established the effects of this adaptation on the perception of gaze direction in subsequently presented male and female test faces. We found that adaptation induced pronounced gaze direction aftereffects, i.e., participants were biased to perceive small gaze deviations to both the left and right as direct. Importantly, however, aftereffects were identical for male and female test faces, showing that the contingency of face sex and gaze direction participants experienced during the adaptation procedure had no effect.

## Introduction

The human face provides us with cues about a person’s identity, age, sex, ethnicity, emotional expression, and current focus of attention (Calder et al., 2011). Our ability to quickly and accurately perceive this information helps us to successfully navigate social interactions.

The efficiency with which our visual system processes the various facial cues is partly owed to *perceptual adaptation* (for a review, see Webster and MacLeod, 2011). The response properties of face-sensitive neurons in the visual system constantly adjust to the specific characteristics of the faces surrounding us. Neural responses to frequently occurring stimulus characteristics are down-regulated, which frees up capacity to respond to novel stimuli and equips us with highly sensitive face discrimination abilities. Behaviorally, the consequences of adaptation are revealed in *aftereffects*, in which perception is systematically biased away from an adapted characteristic. For instance, adaptation to faces with expanded features will bias participants to perceive a subsequently presented undistorted face as slightly compressed. Conversely, after adaptation to compressed faces they will be biased to perceive the same undistorted face as expanded (face distortion aftereffect, Webster and MacLin, 1999).

Aftereffects have been referred to as the “psychologist’s microelectrode” (Frisby, 1980) because they can provide insight in the neuronal organization of the visual system through non-invasive behavioral experiments. Adaptation paradigms have therefore been enthusiastically used to study the face perception system. This research has revealed that the system adapts to variations in practically any facial signal, including identity (Leopold et al., 2001; Rhodes and Jeffery, 2006), sex (Webster et al., 2004; Kovacs et al., 2006; Pond et al., 2013), age (Schweinberger et al., 2010; O’Neil et al., 2014), ethnicity (Webster et al., 2004), emotional expression (Webster et al., 2004), attractiveness (Rhodes et al., 2003; Hayn-Leichsenring et al., 2013), and gaze direction (Jenkins et al., 2006; Seyama and Nagayama, 2006), suggesting that the face perception system contains neural entities that preferentially code, and selectively adapt to, each of these characteristics.

In *contingent face aftereffects* adaptation to opposite characteristics occurs simultaneously for different face categories. This was first described by Rhodes et al. (2004) who used a contingent adaptation paradigm to investigate whether distinct mechanisms code upright and inverted faces. They presented participants with a sequence of alternating faces that were distorted in opposite directions depending on their orientation, for instance expanded upright faces and contracted inverted faces, and studied the aftereffects for subsequently presented undistorted test faces presented in both orientations. The rationale was that if the same neural populations coded upright and inverted faces, the effects of the opposite adaptation distortions, expansion and contraction, should cancel out, and no aftereffect should be observed. However, if distinct populations coded upright and inverted faces, the adaptation procedure should induce separate opposite aftereffects for faces in both orientations. This is indeed what was found. Adaptation to upright expanded and inverted contracted faces induced aftereffects of perceived contraction in upright faces and perceived expansion in inverted faces, indicating that distinct neural populations code upright and inverted faces, and that both of these populations are sensitive toward structural changes on a compressed-expanded dimension (for related findings, see Watson and Clifford, 2006).

Since this initial report, various other studies have used contingent aftereffect paradigms to explore the neural organization of the face perception system. For instance, Little et al. (2005) investigated whether simultaneous opposite aftereffects could also be induced for male and female faces. In separate experiments, they presented participants with male and female faces that systematically differed with respect to their eye spacing, identity, or sexual dimorphism. Sex-contingent opposite aftereffects were found for all of these characteristics. Moreover, other studies have now demonstrated simultaneous opposite distortion aftereffects (Jaquet and Rhodes, 2008), expression aftereffects (Bestelmeyer et al., 2010), and age aftereffects (Schweinberger et al., 2010) for male and female faces. In combination, these studies provide strong evidence that separate neural populations code male and female faces, and that each of these sex-specific populations is sensitive to variations in eye spacing, identity, sexual dimorphism, configuration, emotional expression, and age.

Simultaneous opposite aftereffects have also been induced for European and African faces (ethnicity-contingent eye-spacing aftereffects, Little et al., 2008), for European and Chinese faces (ethnicity-contingent face distortion aftereffects, Jaquet et al., 2008), and for East Asian and African faces (ethnicity-contingent emotional expression aftereffects, Bestelmeyer et al., 2008). Moreover, contingent aftereffects have been described for human and monkey faces (species-contingent eye-spacing aftereffects, Little et al., 2008), for children’s and adult faces (age-contingent eye-spacing aftereffects, Little et al., 2008), and for different familiar face identities (identity-contingent distortion aftereffects, Rooney et al., 2012).

In fact, simultaneous opposite aftereffects have now been reported for so many different combinations of face characteristics that it seems important to pause and question whether they can really provide the proposed level of insight into the structure of the face perception system. Ultimately, it does not seem particularly parsimonious to assume that the face perception system is subdivided to such an extreme extent, i.e., with every possible face category basically possessing its own little face perception subsystem. Is it indeed realistic that information such as the eye spacing, identity, sexual dimorphism, configuration, emotional expression, and age is coded separately for male and female faces? Is it plausible that we have subsets of face-sensitive neurons that almost exclusively respond to Caucasian, East Asian, and African faces and that each of these subsets also selectively codes the eye spacing, configuration, and emotional expression of these faces? Is the eye spacing of children’s and adult faces really coded in distinct channels? In short, can contingent aftereffects indeed be meaningfully interpreted as indicating pre-existing response selectivity toward secondary dimensions in an adapted channel?

A possible alternative explanation is that simultaneous opposite aftereffects reflect a “selectivity” that is only generated during the adaptation procedure, for instance through associative or probabilistic learning. It has been suggested earlier that associative learning might play a role in simultaneous opposite aftereffects patterns (e.g., Murch, 1976). So far, such learning accounts have mostly been discussed for contingent aftereffects in the perception of simple stimulus attributes, such as the McCollough effect, however, they might also explain contingent face aftereffects. In the McCollough effect adaptation to alternating grids of black-and-green vertical bars and black-and-red horizontal bars induces orientation-contingent color aftereffects in the perception of black-and-white grids. Specifically, participants experience the white bars in vertical grids as red, and the white bars in horizontal grids as green. Just like contingent face aftereffects, the McCollough effect was initially suggested to indicate that cells that selectively code for local orientation are also sensitive to color (McCollough, 1965). However, other accounts have suggested that there might be an associative basis to the effect. Specifically, the orientation of the lined grid might act as a conditioned stimulus whereas color represents an unconditioned stimulus and the color aftereffect an unconditioned response. In such a scenario, pairing the conditioned and unconditioned stimulus might induce a conditioned response, a color aftereffect that depends on the orientation of the grid (Murch, 1976; Siegel et al., 1992; Allan and Siegel, 1997).

It is generally conceivable that such associative mechanisms also underlie simultaneous opposite aftereffect patterns in contingent face adaptation paradigms, at least under certain conditions. Prior research has shown that face aftereffects cannot be made contingent on just any physically dissociable categorical stimulus, such as color (Yamashita et al., 2005; Little et al., 2011), unless participants associate these with socially meaningful categorical labels (such as introvert vs. extravert, see Little et al., 2011). This finding might suggests that only visually derivable meaningful social categories can potentially serve as conditioned stimuli (see also, Bestelmeyer et al., 2008). For instance, in sex-contingent emotional expression aftereffects (Bestelmeyer et al., 2010) the sex of the adaptor face might act as a conditioned stimulus, whereas its emotional expression represents an unconditioned stimulus and the expression aftereffect an unconditioned response. Importantly, if contingent face aftereffects were indeed caused by such associative learning mechanisms, it should be possible to make practically any established face aftereffect contingent on any other categorical face characteristic that can serve as a conditioned stimulus. For instance, considering the number and variety of face aftereffects that have been found to be contingent on face sex, one might claim that face sex is a particularly efficient conditioned stimulus and that any face aftereffect might be made “contingent” on it. However, in the present paper we show that this is not the case, and present clear evidence that, unlike so many other face characteristics, gaze direction aftereffects are not contingent on face sex.

Simple gaze direction aftereffects have been well established and often replicated (Jenkins et al., 2006; Seyama and Nagayama, 2006; Schweinberger et al., 2007; Calder et al., 2008; Kloth and Schweinberger, 2008, 2010; Rhodes et al., 2015). Adaptation to faces consistently looking in one direction, for instance 25° to the right, induces strong aftereffects in the perception of gaze in subsequently presented test faces. Specifically, faces with smaller gaze deviations in the adapted direction, e.g., 5° or 10° to the right, are typically falsely perceived to be looking straight at the observer (Jenkins et al., 2006; Seyama and Nagayama, 2006; Schweinberger et al., 2007; Kloth and Schweinberger, 2008, 2010).

**FIGURE 1:**
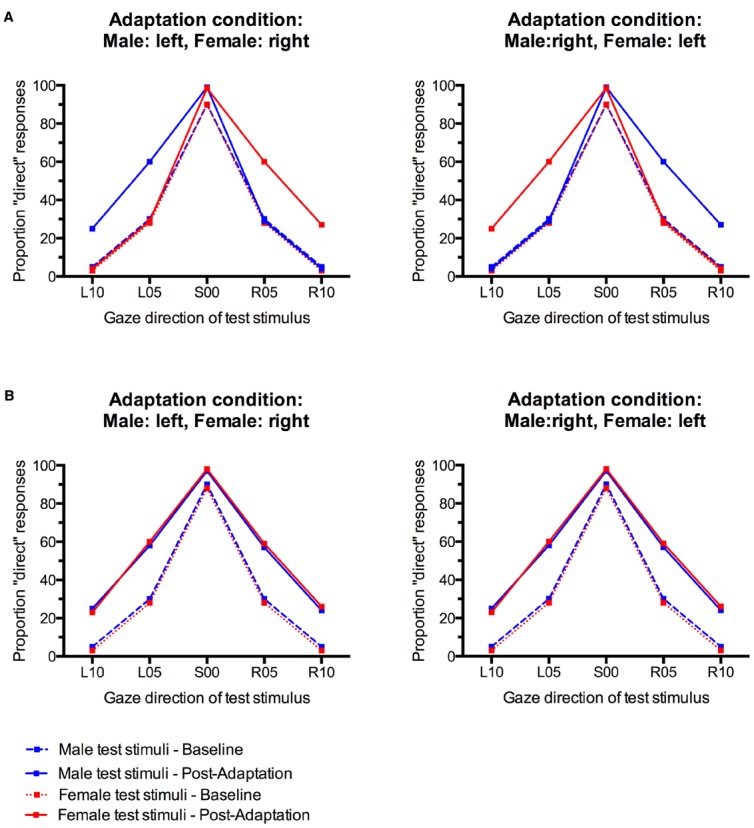
**(A)** Pattern of possible results that would indicate sex-contingency of gaze direction aftereffects. The proportion of “direct” responses only increases for test faces with the same gaze direction as their sex-congruent adaptor. **(B)** Pattern of possible results indicating absence of sex-contingency of gaze direction aftereffects. Irrespective of the adaptation condition and the sex of the test face, there is a general increase in “direct” responses to all test faces.

Here, we studied whether gaze direction aftereffects can be made contingent on face sex. We tested two groups of participants, the first of which adapted to male faces with leftward gaze and female faces with rightward gaze. The second group adapted to male faces with rightward gaze and female faces with leftward gaze. Sex-contingent gaze direction aftereffects would be indicated by different gaze direction aftereffect patterns for male and female test faces between the two groups of participants. The first group would be expected to falsely categorize male faces with left gaze and female faces with right gaze as looking straight ahead. For the second group, however, the opposite pattern would be predicted, resulting in incorrect perceptions of direct gaze from male faces with right gaze and female faces with left gaze (Figure 1A).

If gaze direction aftereffects are not contingent on face sex, alternating adaptation to male and female faces with leftward and rightward gaze should reveal a similar pattern of results for male and female faces in both groups of participants. However, unlike for face distortion aftereffects (Rhodes et al., 2004), the absence of sex-contingency would *not* be indicated by an overall absence of significant gaze direction aftereffects. This is because gaze direction is neurally coded in a non-opponent multichannel system that consists of at least three channels, one primarily responsive to leftward gaze, one primarily responsive to rightward gaze, and a third one primarily sensitive to direct gaze (Calder et al., 2008). This organization makes gaze aftereffects ideally suited to be studied in a contingent adaptation paradigm. Even in the absence of sex contingency, alternating left and right adaptation should lead to a bias in gaze direction perception, characterized by an increase in incorrect classifications of small gaze deviations to the left and right as direct for test faces of both sexes (Figure 1B).

## Materials and Methods

### Participants

Thirty-six Caucasian participants (six men, 18–31 years, *M* = 22, SD = 3) contributed data. All participants reported normal or corrected-to-normal vision and were naïve to the purposes of the study. This study was carried out in accordance with the ethical guidelines of the Declaration of Helsinki and was approved by the ethics committee of the University of Jena. All participants gave written informed consent in accordance with the Declaration of Helsinki and were debriefed after completing the study.

### Stimuli

Test stimuli were color photographs of six male and six female young Caucasian adults with neutral emotional expression taken from earlier research (Jenkins et al., 2006). Each model posed at gaze angles of 10° left (L10), 5° left (L05), direct (S00), 5° right (R05), and 10° right (R10; all directions from the observer’s point of view). Photos of the same 12 models gazing 25° left (L25) and 25° right (R25) were used as adaptation stimuli. All faces were presented in a black elliptical mask. Test stimuli measured 13 cm × 7.5 cm and adaptation stimuli measured 19 cm × 11 cm. A constant viewing distance of ∼87 cm was ensured by using a chin rest.

### Procedure

**FIGURE 2:**
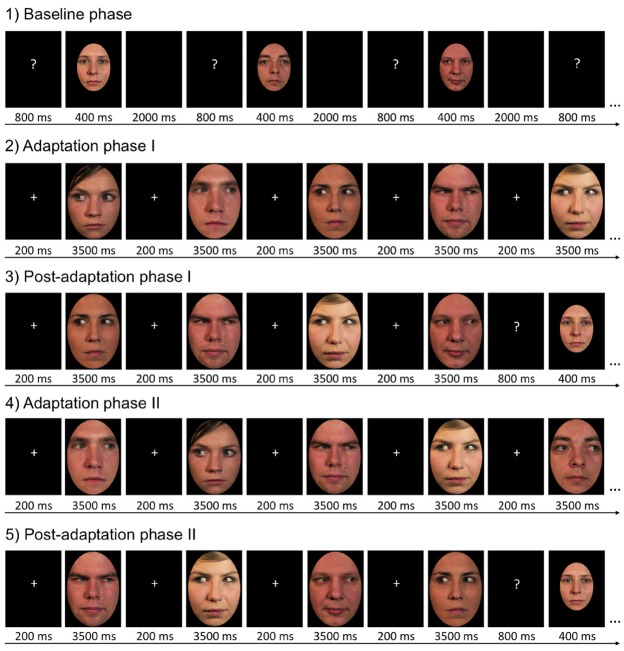
**Trial procedure in the five stages of the experiment.** This example illustrates trials as seen by participants who adapted to female faces with left gaze and male faces with right gaze. The other half of the participants adapted to male faces with left gaze and female faces with right gaze. Please note that the depicted stimulus identities are different from those shown in the actual experiment.

The experiment consisted of five consecutive phases (Figure 2). The *baseline phase* was identical for all participants and served to establish their general ability to determine the gaze direction of the test faces without prior adaptation. Sixty test faces (12 identities × 5 gaze directions) were presented twice in random order. Participants indicated for each face whether it showed left, direct or right gaze direction by pressing one of three labeled response keys. In each trial, a question mark was first presented (800 ms), was then replaced by the test face (400 ms), and followed by a blank screen (2000 ms) during which participants responded. After half of the trials, participants were given a self-paced break. The baseline phase took 6.5 min to complete.

After the baseline phase, participants underwent an adaptation phase and a post-adaptation phase, followed by a second adaptation phase and a second post-adaptation phase. Participants were randomly assigned to one of two adaptation conditions, which were characterized by different adaptor sex/gaze direction contingencies. Half of the participants (*N* = 18, three men, 19–31 years, *M* = 22 ± 3.0) always adapted to male faces with leftward gaze and female faces with rightward gaze, the other half of the participants (*N* = 18, three men, 18–31 years, *M* = 22 ± 3.8) always adapted to male faces with rightward gaze and female faces with leftward gaze. In the *first adaptation phase* participants were presented with three consecutive runs of twelve adaptation stimuli each, presented in pseudo-randomized order. Half of the adaptation stimuli displayed left gaze direction, the other half displayed right gaze direction. Male and female stimuli were presented alternatingly and faces of the same sex always had the same gaze direction. Depending on the adaptation condition participants had been assigned to, all male faces displayed gaze averted 25° to the left and all female faces displayed gaze averted 25° to the right, or *vice versa*. For half of the participants in each adaptation condition, the first adaptation phase started with a male face and ended with a female face, for the other half of participants the first adaptation phase started with a female face and ended with a male face. Exposure duration was 3500 ms for each adaptation stimulus, with an inter-stimulus interval of 200 ms. The adaptation block had a total duration of about 2 min. There was no task associated with this phase, participants were simply asked to passively view the adaptors.

This first adaptation phase was immediately followed by the *first post-adaptation phase* during which participants were again asked to determine the gaze direction of test stimuli. In general, the procedure of the post-adaptation phase was largely equivalent to the baseline phase. The critical difference was that each test stimulus was preceded by four consecutive top-up adaptors (3500 ms each) presented before the question mark (800 ms) and the test face (400 ms) to ensure consistently high levels of adaptation throughout the entire post-adaptation phase. Top-up stimuli also alternated in sex and began with the same sex as the adaptation sequence in the first adaptation phase. Neither of the top-up adaptation stimuli carried the same identity as the following test face. There were 60 trials in the first post-adaptation block and participants were given self-paced breaks after every 20 trials. The completion of the first post-adaptation phase took about 16 min.

To avoid inducing systematic biases toward the gaze direction (and sex) of the top-up face that was presented immediately before the test face, the first post-adaptation phase was followed by another adaptation sequence. This *second adaptation phase* differed from the first one only with respect to the order in which adaptors of the different sexes were presented. Participants who had adapted to a sequence of faces beginning with a male face in the first adaptation phase, were presented with a sequence of faces that started with a female face in the second adaptation phase. In the following *second post-adaptation phase*, the order of male and female top-up stimuli was adjusted accordingly. Importantly, the contingency of adaptor sex and adaptor gaze direction in the second adaptation and post-adaptation phase was identical to the one participants had experienced in the first adaptation and post-adaptation phase. The reversal of the order in which male and female adaptors were presented simply ensured that across the whole experiment, test faces were equally likely to be immediately preceded by a male or a female adaptor. Overall, the experiment took 45–50 min to complete.

## Results

**FIGURE 3:**
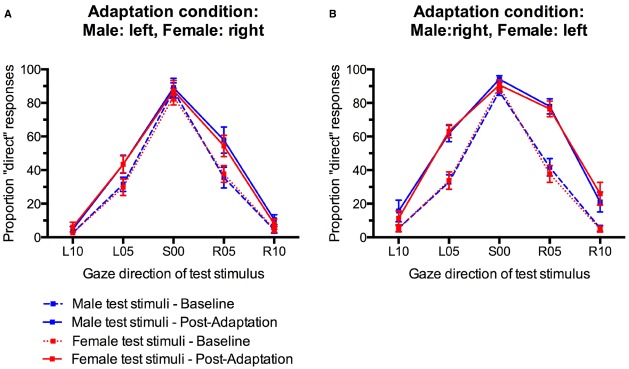
**Percentage of “direct” classifications in response to male and female faces before and after gaze adaptation. (A)** Adaptation to male faces with left gaze and female faces with right gaze. **(B)** Adaptation to male faces with right gaze and female faces with left gaze. Error bars indicate SEMs.

Gaze direction adaptation typically leads to an increased tendency to falsely classify gaze in the adapted direction as direct (Jenkins et al., 2006; Schweinberger et al., 2007; Calder et al., 2008; Kloth and Schweinberger, 2008). Gender-contingent gaze adaptation would therefore be revealed by a pattern of increased “direct” classifications that differed systematically between male and female test faces. Specifically, there should be a selective increase in “direct” responses only for those test faces that look in the same direction as the sex-congruent adaptors. For instance, participants who adapted to male stimuli with left gaze and female stimuli with right gaze would be expected to show a selective increase in “direct” responses only to male test faces with left gaze and female test faces with right gaze, but not to male faces with right gaze and female faces with left gaze (see Figure 1A). The empirical data do not suggest such a pattern (Figure 3). Instead, participants seem to generally show aftereffects in the perception of both left and right gaze direction, indicated by an overall increase in “direct” classifications, irrespective of the sex of the test face (cf., Figure 1B).

**FIGURE 4:**
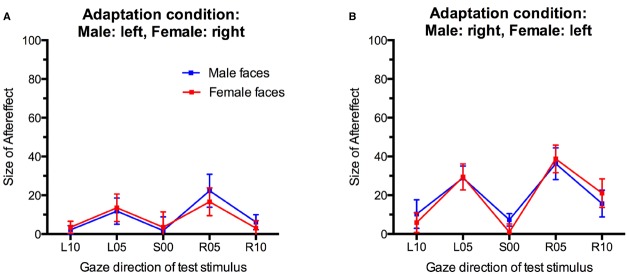
**Gaze direction aftereffects for the five different gaze directions observed for male and female test stimuli. (A)** Adaptation to male faces with left gaze and female faces with right gaze. **(B)** Adaptation to male faces with right gaze and female faces with left gaze. Error bars indicate SEMs.

For statistical analysis, we obtained a measure for the size of the gaze direction aftereffect by subtracting the percentage of “direct” classifications in the pre-adaptation baseline from the percentage of “direct” classifications after adaptation (collapsed across the first and second post-adaptation phase) for each participant and each condition. Positive scores indicate an increase in direct classifications after adaptation while negative scores indicate a decreased in direct classifications compared to baseline (Figure 4). These aftereffect scores were entered into an analysis of variance (ANOVA) with Sex of test face (male, female) and Gaze direction of test face (L10, L05, S00, R05, R10) as within-participants factors and Adaptation Condition (Male:left, Female:right; Male:right, Female:left) as a between-participants factor.

There was a main effect of Gaze Direction, *F*(4,136) = 10.86, *p* < 0.001, ${\text{η}}_{p}^{2}$ = 0.24. Single-sample *t*-tests indicated that aftereffects were significantly larger than 0 for test faces with 5° leftward gaze, *t*(35) = 4.48, *p* < 0.001, *d* = 1.49, 5° rightward gaze, *t*(35) = 5.29, *p* < 0.001, *d* = 1.76, and 10° rightward gaze, *t*(35) = 2.89, *p* = 0.007, *d* = 0.98. Aftereffects for faces with 10° leftward gaze and direct gaze were not significant, *t*(35) = 1.74, *p* = 0.09, *d* = 0.59 and *t*(35) = 0.80, *p* = 0.43, *d* = 0.27, respectively. Aftereffects were larger for test stimuli with 5° gaze deviation than with 10° gaze deviation, *t*(35) = 3.94, *p* < 0.001, *d* = 0.66, and *t*(35) = 4.10, *p* < 0.001, *d* = 0.68, for the comparison of aftereffects for L05 vs. L10 and R05 vs. R10 test stimuli, respectively. Additionally, significantly larger aftereffects were observed for R10 than L10 stimuli, *t*(35) = 3.04, *p* < 0.01, *d* = 0.51.

Importantly, while the present paradigm clearly induced significant gaze direction aftereffects, these did not differ significantly for male and female faces in the two different adaptation conditions, *F*(4,136) = 1.49, *p* = 0.21, ${\text{η}}_{p}^{2}$ = 0.04, for the interaction of Sex of test face, Gaze Direction of test face, and Adaptation Condition. The main effect of Adaptation Condition was not significant, *F*(1,34) = 2.58, *p* = 0.12, ${\text{η}}_{p}^{2}$ = 0.07. There were no other significant effects (all *F*s < 1, all *p*s > 0.40).

**FIGURE 5:**
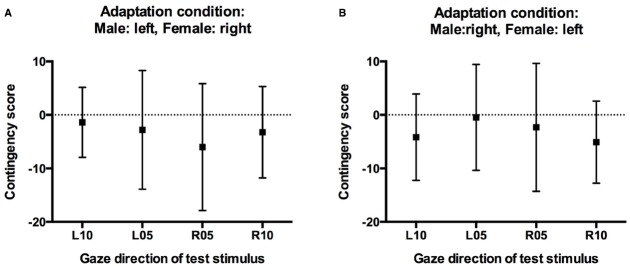
**Contingency scores. (A)** Contingency scores for participants who adapted to male faces with left gaze and female faces with right gaze. **(B)** Contingency scores for participants who adapted to male faces with right gaze and female faces with left gaze. Error bars indicate 95% confidence intervals.

Critically, the absence of a significant three-way interaction in the above analysis cannot be taken as conclusive evidence against contingency. In a final step, therefore, we calculated contingency scores for test stimuli with averted gaze, separately for participants in the two adaptation conditions based on the predicted contingency pattern. For participants who had adapted to male faces with leftward gaze and female faces with rightward gaze, one would predict a selective increase in “direct” classifications relative to baseline only for male faces with 5° and 10° leftward gaze, but not for female faces with 5° and 10° leftward gaze. Conversely, one would predict a selective increase in “direct” classifications only for female faces with 5° and 10° rightward gaze, but not for male faces with 5° and 10° rightward gaze. For participants in this adaptation condition, contingency scores for the leftward gaze conditions were therefore calculated by subtracting the increase in direct responses made to female faces with leftward gaze after adaptation relative to baseline (i.e., the unpredicted aftereffect scores for female faces) from the increase in direct responses made to male faces with leftward gaze after adaptation relative to baseline (i.e., the predicted aftereffect scores for male faces). Conversely, contingency scores for the rightward gaze conditions were calculated by subtracting the unpredicted increase in direct classifications made to male faces with rightward gaze from the predicted increase in direct classifications made to female faces with rightward gaze. For participants who had adapted to male faces with rightward gaze and female faces with leftward gaze, contingency scores were calculated accordingly. Positive contingency scores therefore indicate a contingent aftereffect pattern, with larger aftereffects for test faces of the same sex as the adaptors looking in the same direction compared to opposite-sex faces, contingency scores around 0 would indicate the absence of such contingencies, i.e., similar aftereffects for test faces of both sexes. Importantly, and confirming the above analyses, contingency scores were close to 0, and 95% confidence intervals included 0 in all conditions (Figure 5). One-sample *t*-tests confirmed that contingency scores were not significantly different from 0 for any gaze direction in either adaptation condition (all *t*s < 1.5, all *p*s > 0.18).

## Discussion

We have shown that adaptation to alternating male and female faces with opposite gaze directions induces significant gaze direction aftereffects that are not contingent on face sex. Instead, test faces of either sex and with either leftward or rightward gaze were more likely to be perceived as looking straight ahead after adaptation than during the baseline phase (cf., Calder et al., 2008). These data indicate that gaze direction aftereffects are not contingent on face sex. Moreover, they suggest that the same neural populations process the gaze direction of male and female faces.

Importantly, the neural coding principles underlying gaze direction perception allow us to interpret this negative finding without having to worry that the absence of sex-contingent gaze direction aftereffects might simply indicate an inefficient adaptation procedure. Gaze direction perception is multichannel coded and therefore alternating left and right adaptation does not cancel out, but induces aftereffects in the perception of both gaze directions (Calder et al., 2008). We replicate this finding here, which demonstrates that our adaptation procedure was effective and produced significant gaze direction aftereffects. Importantly though, these aftereffects are indistinguishable for male and female faces, indicating that they are completely unaffected by the contingency between gaze direction and face sex present in the adaptation sequence.

Our findings suggest that contingency is not a general property of face aftereffects, despite having been observed for various different combinations of face dimensions (e.g., Rhodes et al., 2004; Little et al., 2005, 2008; Fox and Barton, 2007; Bestelmeyer et al., 2008; Schweinberger et al., 2010). Moreover, our data also suggest that the many sex-contingent opposite face aftereffects reported previously are unlikely to simply have resulted from associative learning during the contingent adaptation sequence. An associative learning account of contingent adaptation (Murch, 1976; Siegel et al., 1992; Allan and Siegel, 1997) would predict that once a stimulus category (e.g., face sex) has been demonstrated to have the potential to work as a conditioned stimulus for a face aftereffect (e.g., emotion aftereffect, eye-spacing aftereffect, age aftereffect), any other established face aftereffect (e.g., gaze direction aftereffect) should be easily made contingent on that category as well. Here we show, that this is not the case, providing some indirect evidence for the traditional assumption that contingent aftereffects indicate pre-existing response selectivity toward a second dimension in an adapted channel.

When considered in combination with earlier studies, our findings suggest that the perception of only some face characteristics is contingent on a second category, whereas the perception of other characteristics seems to be more general and independent of other categories, as indicated by an absence of contingent aftereffects. The variety of published reports on contingent face aftereffects (Rhodes et al., 2004; Little et al., 2005, 2008; Fox and Barton, 2007; Bestelmeyer et al., 2008; Schweinberger et al., 2010) and the lack of studies reporting an absence of contingent aftereffects, might suggest that more face signals are processed interactively rather than independently. However, the lack of studies reporting an absence of contingent aftereffects might also reflect a publication bias due to the relative difficulty to publish negative findings.

Interestingly, earlier research has shown that *eye spacing* aftereffects are contingent on face sex (Little et al., 2005), suggesting that configural changes in the eye region of male and female faces are processed by different neural populations. This finding may be related to evidence that face identity is coded in a norm-based manner, which is largely specific to the sex of the face (Jaquet and Rhodes, 2008). In other words, separate sex-specific prototypes seem to be used for the identification of male and female faces, and these can be simultaneously adapted in opposite directions. The fact that different norms exist for identity processing of male and female faces, but that there is no evidence for similarly sex-specific gaze processing mechanisms confirms the idea that, despite evidence for integrative processing of various signals in the face, at least some characteristics are processed independently (Bruce and Young, 1986; Haxby et al., 2000).

Earlier research studying the interdependence of the processing of different facial signals provides converging evidence that some of them might be processed more interdependently than others. For instance, using a selective attention paradigm to study the interdependency of identity and expression processing, Schweinberger and Soukup (1998) reported that variations in identity slowed down classifications of emotional expression, but that variations in emotional expressions did not affect classifications of identity. Interestingly, more recent research has replicated parts of this asymmetrical interference in a contingent aftereffect paradigm: In line with the observations of Schweinberger and Soukup (1998), emotional expression aftereffects have been found to be contingent on face identity (Fox and Barton, 2007). Moreover, while we are not aware of any research that would have investigated whether identity aftereffects are also contingent on emotional expressions, there is at least evidence that identity aftereffect completely transfer across different emotional expression (Fox et al., 2008).

Our data suggest that gaze direction perception is largely independent of face sex. Interestingly, there is substantial evidence from various other studies that gaze direction perception *can* generally be affected by other facial signals. For instance, the emotional expression of a face (Ganel et al., 2005; Graham and LaBar, 2007; Ewbank et al., 2009) as well as its attractiveness (Kloth et al., 2011) have both been found to affect the perceived gaze direction of a face. There is also evidence that information from the eye region can be diagnostic about a face’s sex and that certain gaze directions can enhance or decrease ratings of masculinity and femininity in faces (Campbell et al., 1996). The present results might be taken to suggest that this influence likely originates from post-perceptual processes such as top-down strategies, for instance self-referential positivity biases (Lobmaier et al., 2008), rather than early perceptual integration (see also, Kloth et al., 2011).

Gaze direction might be rather unique compared to other facial signals, in that it might generally be more independent of the overall structure of the face than any other face signal (cf., Rhodes et al., 2015). There is evidence that local shape and luminance information play an important role in the perception of gaze direction, suggesting that gaze direction perception relies more on relatively simple cues processed on low-levels of the visual system (Anstis et al., 1969; Ando, 2002, 2004; Jenkins, 2007) than other face information. Having said that, it is important to keep in mind that gaze direction aftereffects clearly do also involve higher levels of the visual system (Jenkins et al., 2006), suggesting that their perception is not solely based on low-level visual processing.

A potential limitation of our research is that we do not have explicit evidence that the male and female adaptor faces used in the present study are generally able to induce sex-contingent aftereffects on other face attributes than gaze direction. However, visual inspection of the stimuli suggests that the faces have perfectly normal sexual dimorphism. Therefore, there is no immediate reason to assume that they would not be able to induce sex-contingent gaze direction aftereffects, if separate gaze-sensitive neural channels existed for male and female faces. We also note that our participant sample was predominantly female, but there is no reason to think that participant sex would affect the potential for sex-contingent aftereffects.

In summary, we used a sex-contingent gaze adaptation paradigm to explore whether, like so many other face aftereffects, gaze direction aftereffects are contingent on face sex. We found significant gaze direction aftereffects in all experimental conditions, however, these were completely unaffected by the contingency of adaptor sex and gaze direction presented during the adaptation sequence. These data suggest that it is rather unlikely that the large variety of sex-contingent face aftereffects reported in earlier work is due solely to associative learning mechanisms during the contingent adaptation sequence. Instead, separate neural populations seem to selectively respond to male and female faces, and separately code signals such as the emotional expression, eye spacing, sexual dimorphism and age of these faces. In contrast to these signals, gaze direction appears to be coded more generally, and independently of the sex of a face.

## Author Contributions

Designed the experiment: NK, SS; data acquisition: NK; data analyses: NK, GR, SS; interpretation of the data: NK, GR, SS; provided materials: NK, SS; wrote the article: NK, GR, SS; proofed/revised the article: NK, GR, SS.

### Conflict of Interest Statement

The authors declare that the research was conducted in the absence of any commercial or financial relationships that could be construed as a potential conflict of interest.

## References

[B1] Allan L. G. Siegel S. (1997). Contingent color aftereffects: reassessing old conclusions. Percept. Psychophys. 59, 129–141. 10.3758/BF03206855 9038415

[B2] Ando S. (2002). Luminance-induced shift in the apparent direction of gaze. Perception 31, 657–674. 10.1068/p3332 12092793

[B3] Ando S. (2004). Perception of gaze direction based on luminance ratio. Perception 33, 1173–1184. 10.1068/p5297 15693663

[B4] Anstis S. M. Mayhew J. W. Morley T. (1969). Perception of where a face or television portrait is looking. Am. J. Psychol. 82, 474–489. 10.2307/1420441 5398220

[B5] Bestelmeyer P. E. G. Jones B. C. DeBruine L. M. Little A. C. Perrett D. I. Schneider A. (2008). Sex-contingent face aftereffects depend on perceptual category rather than structural encoding. Cognition 107, 353–365. 10.1016/j.cognition.2007.07.018 17870064

[B6] Bestelmeyer P. E. G. Jones B. C. DeBruine L. M. Little A. C. Welling L. L. M. (2010). Face aftereffects suggest interdependent processing of expression and sex and of expression and race. Vis. Cogn. 18, 255–274. 10.1080/13506280802708024

[B7] Bruce V. Young A. (1986). Understanding face recognition. Br. J. Psychol. 77, 305–327. 10.1111/j.2044-8295.1986.tb02199.x 3756376

[B8] Calder A. J. Jenkins R. Cassel A. Clifford C. W. G. (2008). Visual representation of eye gaze is coded by a nonopponent multichannel system. J. Exp. Psychol. Gen. 137, 244–261. 10.1037/0096-3445.137.2.244 18473657

[B9] Calder A. J. Rhodes G. Johnson M. Haxby J. V. (eds). (2011). The Oxford Handbook of Face Perception. New York: Oxford University Press.

[B10] Campbell R. Wallace S. Benson P. J. (1996). Real men don’t look down: direction of gaze affects sex decisions on faces. Vis. Cogn. 3, 393–412. 10.1080/135062896395643

[B11] Ewbank M. P. Jennings C. Calder A. J. (2009). Why are you angry with me? Facial expressions of threat influence perception of gaze direction. J. Vis. 9, 16. 10.1167/9.12.16 20053107

[B12] Fox C. J. Barton J. J. S. (2007). What is adapted in face adaptation? The neural representations of expression in the human visual system. Brain Res. 1127, 80–89. 10.1016/j.brainres.2006.09.104 17109830

[B13] Fox C. J. Oruc I. Barton J. J. S. (2008). It doesn’t matter how you feel. The facial identity aftereffect is invariant to changes in facial expression. J. Vis. 8, 11. 10.1167/8.3.11 18484817

[B14] Frisby J. P. (1980). Seeing: Illusion, Mind and Brain. Oxford: Oxford University Press.

[B15] Ganel T. Goshen-Gottstein Y. Goodale M. A. (2005). Interactions between the processing of gaze direction and facial expression. Vision Res. 45, 1191–1200. 10.1016/j.visres.2004.06.025 15707927

[B16] Graham R. LaBar K. S. (2007). Garner interference reveals dependencies between emotional expression and gaze in face perception. Emotion 7, 296–313. 10.1037/1528-3542.7.2.296 17516809

[B17] Haxby J. V. Hoffman E. A. Gobbini M. I. (2000). The distributed human neural system for face perception. Trends Cogn. Sci. 4, 223–233. 10.1016/S1364-6613(00)01482-0 10827445

[B18] Hayn-Leichsenring G. U. Kloth N. Schweinberger S. R. Redies C. (2013). Adaptation effects to attractiveness of face photographs and art portraits are domain-specific. Iperception 4, 303–316. 10.1068/i0583 24349690 PMC3859548

[B19] Jaquet E. Rhodes G. (2008). Face aftereffects indicate dissociable, but not distinct, coding of male and female faces. J. Exp. Psychol. Hum. Percept. Perform. 34, 101–112. 10.1037/0096-1523.34.1.101 18248142

[B20] Jaquet E. Rhodes G. Hayward W. G. (2008). Race-contingent aftereffects suggest distinct perceptual norms for different race faces. Vis. Cogn. 16, 734–753. 10.1080/13506280701350647

[B21] Jenkins R. (2007). The lighter side of gaze perception. Perception 36, 1266–1268. 10.1068/p5745 17972488

[B22] Jenkins R. Beaver J. D. Calder A. J. (2006). I thought you were looking at me—direction-specific aftereffects in gaze perception. Psychol. Sci. 17, 506–513. 10.1111/j.1467-9280.2006.01736.x 16771801

[B23] Kloth N. Altmann C. S. Schweinberger S. R. (2011). Facial attractiveness biases the perception of eye contact. Q. J. Exp. Psychol. 64, 1906–1918. 10.1080/17470218.2011.587254 21756185

[B24] Kloth N. Schweinberger S. R. (2008). The temporal decay of eye gaze adaptation effects. J. Vis. 8, 4. 10.1167/8.11.4 18831598

[B25] Kloth N. Schweinberger S. R. (2010). Electrophysiological correlates of eye gaze adaptation. J. Vis. 10, 17. 10.1167/10.12.17 21047749

[B26] Kovacs G. Zimmer M. Banko E. Harza I. Antal A. Vidnyanszky Z. (2006). Electrophysiological correlates of visual adaptation to faces and body parts in humans. Cereb. Cortex 16, 742–753. 10.1093/cercor/bhj020 16120795

[B27] Leopold D. A. O’Toole A. J. Vetter T. Blanz V. (2001). Prototype-referenced shape encoding revealed by high-level after effects. Nat. Neurosci. 4, 89–94. 10.1038/82947 11135650

[B28] Little A. C. DeBruine L. M. Jones B. C. (2005). Sex-contingent face after-effects suggest distinct neural populations code male and female faces. Proc. R. Soc. B Biol. Sci. 272, 2283–2287. 10.1098/rspb.2005.3220 16191641 PMC1560190

[B29] Little A. C. DeBruine L. M. Jones B. C. (2011). Category-contingent face adaptation for novel colour categories: contingent effects are seen only after social or meaningful labelling. Cognition 118, 116–122. 10.1016/j.cognition.2010.09.011 21040911

[B30] Little A. C. DeBruine L. M. Jones B. C. Waitt C. (2008). Category contingent aftereffects for faces of different races, ages and species. Cognition 106, 1537–1547. 10.1016/j.cognition.2007.06.008 17707364

[B31] Lobmaier J. S. Tiddeman B. P. Perrett D. I. (2008). Emotional expression modulates perceived gaze direction. Emotion 8, 573–577. 10.1037/1528-3542.8.4.573 18729587

[B32] McCollough C. (1965). Color adaptation of edge-detectors in the human visual system. Science 149, 1115–1116. 10.1126/science.149.3688.1115 17737844

[B33] Murch G. M. (1976). Classical conditioning of McCollough effect: temporal parameters. Vision Res. 16, 615–619. 10.1016/0042-6989(76)90008-0 960585

[B34] O’Neil S. F. Mac A. Rhodes G. Webster M. A. (2014). Adding years to your life (or at least looking like it): a simple normalization underlies adaptation to facial age. PLoS ONE 9:e116105. 10.1371/journal.pone.0116105 25541948 PMC4277445

[B35] Pond S. Kloth N. McKone E. Jeffery L. Irons J. Rhodes G. (2013). Aftereffects support opponent coding of face gender. J. Vis. 13, 16. 10.1167/13.14.16 24361588

[B36] Rhodes G. Jeffery L. (2006). Adaptive norm-based coding of facial identity. Vision Res. 46, 2977–2987. 10.1016/j.visres.2006.03.002 16647736

[B37] Rhodes G. Jeffery L. Watson T. L. Clifford C. W. G. Nakayama K. (2003). Fitting the mind to the world: face adaptation and attractiveness aftere-ffects. Psychol. Sci. 14, 558–566. 10.1046/j.0956-7976.2003.psci_1465.x 14629686

[B38] Rhodes G. Jeffery L. Watson T. L. Jaquet E. Winkler C. Clifford C. W. G. (2004). Orientation-contingent face aftereffects and implications for face-coding mechanisms. Curr. Biol. 14, 2119–2123. 10.1016/j.cub.2004.11.053 15589154

[B39] Rhodes G. Pond S. Burton N. Kloth N. Jeffery L. Bell J. (2015). How distinct is the coding of face identity and expression? Evidence for some common dimensions in face space. Cognition 142, 123–137. 10.1016/j.cognition.2015.05.012 26036924

[B40] Rooney B. Keyes H. Brady N. (2012). Shared or separate mechanisms for self-face and other-face processing? Evidence from adaptation. Front. Psychol. 3:66. 10.3389/fpsyg.2012.00066 22408633 PMC3296062

[B41] Schweinberger S. R. Kloth N. Jenkins R. (2007). Are you looking at me? Neural correlates of gaze adaptation. Neuroimage 18, 693–696. 10.1097/wnr.0b013e3280c1e2d2 17426601

[B42] Schweinberger S. R. Soukup G. R. (1998). Asymmetric relationships among perceptions of facial identity, emotion, and facial speech. J. Exp. Psychol. Hum. Percept. Perform. 24, 1748–1765. 10.1037/0096-1523.24.6.1748 9861721

[B43] Schweinberger S. R. Zaske R. Walther C. Golle J. Kovacs G. Wiese H. (2010). Young without plastic surgery: perceptual adaptation to the age of female and male faces. Vision Res. 50, 2570–2576. 10.1016/j.visres.2010.08.017 20800608

[B44] Seyama J. Nagayama R. S. (2006). Eye direction aftereffect. Psychol. Res. 70, 59–67. 10.1007/s00426-004-0188-3 15378364

[B45] Siegel S. Allan L. G. Eissenberg T. (1992). The associative basis of contingent color aftereffects. J. Exp. Psychol. Gen. 121, 79–94. 10.1037/0096-3445.121.1.79 1534349

[B46] Watson T. L. Clifford C. W. G. (2006). Orientation dependence of the orientation-contingent face aftereffect. Vision Res. 46, 3422–3429. 10.1016/j.visres.2006.03.026 16723149

[B47] Webster M. A. Kaping D. Mizokami Y. Duhamel P. (2004). Adaptation to natural facial categories. Nature 428, 557–561. 10.1038/nature02420 15058304

[B48] Webster M. A. MacLeod D. I. A. (2011). Visual adaptation and face perception. Philos. Trans. R. Soc. B Biol. Sci. 366, 1702–1725. 10.1098/rstb.2010.0360 21536555 PMC3130378

[B49] Webster M. A. MacLin O. H. (1999). Figural aftereffects in the perception of faces. Psychon. Bull. Rev. 6, 647–653. 10.3758/BF03212974 10682208

[B50] Yamashita J. A. Hardy J. L. De Valois K. K. Webster M. A. (2005). Stimulus selectivity of figural aftereffects for faces. J. Exp. Psychol. Hum. Percept. Perform. 31, 420–437. 10.1037/0096-1523.31.3.420 15982123

